# Editorial: Applications of herbal medicine to control chronic kidney disease: Volume II

**DOI:** 10.3389/fphar.2022.1086049

**Published:** 2022-11-28

**Authors:** Jianping Chen, Karl W. K. Tsim, Ying-Yong Zhao

**Affiliations:** ^1^ Shenzhen Key Laboratory of Hospital Chinese Medicine Preparation, Shenzhen Traditional Chinese Medicine Hospital, The Fourth Clinical Medical College of Guangzhou University of Chinese Medicine, Shenzhen, China; ^2^ Division of Life Science and Center for Chinese Medicine, The Hong Kong University of Science and Technology, Hong Kong SAR, China; ^3^ Faculty of Life Science and Medicine, Northwest University, Xi’an, Shaanxi, China

**Keywords:** chronic kidney disease, herbal medicine, mechanism, active component, safety usage

## Introduction

Chronic kidney disease (CKD) is a leading cause of life lost worldwide, representing a global public health burden (Chen et al., 2019). The pathophysiological mechanisms leading to progression of CKD include parenchymal cell loss, chronic inflammation, fibrosis and reduced regenerative capacity of kidney (Ruiz-Ortega et al., 2020; Speer et al., 2022). The complications of CKD, e.g., anemia and cardiovascular disease, are associated with increased risks of death (Fishbane and Spinowitz, 2018; Ravid et al., 2021). The prevalence and mortality of CKD are collectively increasing, illustrating the need for efficacious therapeutic approaches. This situation therefore calls for novel therapies for CKD and its complications to prevent disease progression and to prevent death. In recent years, herbal medicine has represented additional therapeutic alternatives in the management and treatment of numerous diseases, and which is attracting greater attention and being applied to stop or reverse CKD progression. In this Research Topic, our goal is to provide a forum to advance research of herbal medicine towards CKD therapies. Twenty-nine contributions covering the listed Research Topics have been collected to this Research Topic.

### Reviews of herbal medicine in the treatment of kidney disease

Wang et al. summarized the theories and treatment strategies of traditional Chinese medicine (TCM) in treating CKD. The strategies and mechanisms of TCM against CKD from different forms of medication, such as usage of single herb (Astragali Radix, roots of *Astragalus membranaceus*), or herbal pairs (Astragali Radix-Angelicae Sinensis Radix Pair), or herbal formula (Zhenwu Tang). TCMs have major role in inhibiting activation of myofibroblasts, infiltration of inflammatory cells, trans-differentiation of epithelial cells to mesenchymal cells, and excessive deposition of extracellular matrix to improve renal fibrosis through various mechanisms. Renal fibrosis, especially tubulointerstitial fibrosis, is the final manifestation of CKD. Yu et al. have summarized the role of TGF-β/Smad signaling pathway in tubulointerstitial fibrosis. Moreover, the authors have pointed out that natural products, including *A. membranaceus*, *Salvia miltiorrhiza*, *Poria cocos* and *Rheum palmatum*, as well as their phytochemicals, i.e., astragaloside IV, salvianolic acid, poricoic acids, are exerting anti-tubulointerstitial fibrosis effects by targeting TGF-β/Smad signaling. Growing evidence has indicated that hyperuricemia is an independent risk factor in development and progression of CKD, and therefore a review by Yang et al. has provided an overview of efficacy and mechanisms of TCM or natural products in hyperuricemia-induced CKD. The authors presented 11 herbs, such as *Smilax china*, *Liriodendron chinense*, *Dendrobium officinale* and *Rhododendron oldhamii*, have renal-protective effects on hyperuricemic nephropathy via lowering serum excessive uric acid level. The mechanisms of which are involved in inhibiting activity of liver xanthine oxidoreductase and/or regulating expressions of renal urate transporters. Podocyte injury is one of common causes of proteinuric kidney diseases. Here, Lin et al. have discussed the potential mechanism of TCM and its active components in protecting podocytes, such as repairing podocyte injury, inhibiting podocyte proliferation, reducing podocyte apoptosis and excretion, maintaining podocyte skeleton structure and upregulating podocyte-related protein expression, supporting the development of new therapeutics against primary podocytosis. In support of this notion, a review by Miao et al. has summed up the applications of TCM such as *Tripterygium wilfordii* and *A. membranaceus* for the treatment of membranous nephropathy, a renal-limited non-inflammatory autoimmune disease in the glomerulus. Wang et al. have summarized the role of metabolic reprogramming in diabetic kidney disease, one of the most common microvascular complications of diabetes mellitus. Furthermore, a meta-analysis of randomized controlled trials was conducted by Fu et al. to clarify the effect of hydroxyl safflower yellow A (from *Carthamus tinctorius*) on oxidative stress and inflammatory response in patients with diabetic kidney disease. Collectively, these review articles are providing valuable references in supporting efficacy and mechanism of herbal medicine in kidney diseases.

### Herbal medicine and its active ingredients for kidney disease

Eleven articles are focused on the usage of single herb and the identification of active ingredients within the herb in treating kidney disease. Li et al. have elucidated the effects and mechanisms of paeoniflorin, one of major bio-actives extracted from roots of *Paeonia lactiflora* on CKD skeletal muscle atrophy. The results have shown that paeoniflorin can improve renal functions, calcium/phosphorus disorders and nutrition indexes in the nephrectomized CKD rats, and the mechanism of which are involved in suppressing oxidative stress and mitochondrial dysfunction, partially through the AMPK/SIRT1/PGC-1α pathway. Astragaloside IV, an active ingredient of *A. membranaceus* was also found to have beneficial role in nephrectomized CKD model (He et al.). The results demonstrated that astragaloside IV attenuated intestinal barrier dysfunction via AKT-GSK3β-β-catenin pathway in peritoneal dialysis. Moreover, formononetin, another major constituent from *A. membranaceus,* has been reported to improve albuminuria, renal tubular injury, and mitochondrial damage in streptozotocin-induced diabetic rats, and the mechanism of which was partly through regulating Sirt1/PGC-1α pathway (Huang et al.). Supporting this notion, a study by Zhang et al. further demonstrated the protective effect of calycosin, an active flavonoid of *A. membranaceus,* on renal ischemia/reperfusion (I/R) injury. Calycosin was found to improve kidney function and to reduce renal inflammation responses, and the NF-κB-mediated inflammatory response via PPARγ/EGR1 pathway was involved. Ding et al. (2022) have found that total extract of flowers of *Abelmoschus manihot* was able to attenuate the uric acid-induced NRK-52E cell injury, mimicking a hyperuricemia condition. The results showed that *A. manihot* protected against uric acid-induced cell injury through anti-pyroptotic effect by downregulating caspase-8/caspase-3/NLPR3 (NOD-like receptor thermal protein domain associated protein 3)/GSDME (gasdermin E) signaling. Another study by Zhang et al. has revealed the therapeutic effect of urolithin A, a major intestinal metabolite of ellagitannins from a family of polyphenols presenting in fruits and nuts, in a fructose-induced hyperuricemic nephropathy mouse model, and the effect of which was involved in impairing STING-NLRP3 axis-mediated inflammatory response via Parkin-dependent mitophagy.

Acute kidney injury (AKI) is one of significant risk factors in development of CKD, and three articles are exploring therapy for preventing AKI or delaying its progression to CKD. First, Dou et al. have explored the protective role of *S. miltiorrhiza* and its bioactive compound tanshinone IIA (TanIIA) in AKI. The authors reported that nuclear receptor family could be the target of *S. miltiorrhiza* in AKI treatment by a prediction from network pharmacology. Furthermore, TanIIA was found to improve cell necrosis proliferation and to reduce renal inflammation by stimulating the expression of pregnane X receptor and inhibiting NF-κB signaling. Another study by Liu et al. investigated the effect of tilianin (acacetin-7-glucoside), a bio-active flavonoid glycoside isolated from various medicinal plants, e.g., *Agastache rugosa,* in I/R-induced AKI mice. The authors found that tilianin could reduce apoptosis after I/R-induced AKI by ERK/EGR1/BCL2L1 pathway. To further support the role of natural products in AKI, Yao et al. have evaluated the therapeutic effect of oroxylin A, an active component of *Scutellaria baicalensis,* in I/R and cisplatin indued-AKI mice. The results showed that oroxylin A ameliorated tubular damage and dramatically decreased serum creatinine and urea nitrogen, and the expressions of renal injury markers (Kim-1, Ngal) in AKI mice, as well as attenuating AKI-to-CKD transition, and which was involved in maintaining PPARα-BNIP3 signaling-mediated mitochondrial homeostasis. In addition to AKI, Li et al. have investigated the protective effects of *Phyllanthus niruri* on calcium oxalate-induce renal injury in mice, a urolithiasis or kidney stones model. Ellagic acid was identified as the active ingredient from *P. niruri* in inhibiting the increased expressions of squalene monooxygenase (SQLE), stearoyl-CoA desaturase (SCD) and 3-Hydroxy-3-Methylglutaryl-CoA Synthase 1 (GMGCS1) in oxalate-induced renal injury in HK-2 cells and mice model. The article by Chen et al. has shown that saponins from *Panax notoginseng* effectively alleviated steroid resistance in methylprednisolone induced steroid-resistant lupus nephritis mouse model through the regulation of lymphocyte-derived exosomes.

Four articles have focused on herbal medicine in a formulated mixture for the treatment of kidney disease. Wang et al. have evaluated the protective effects of *Rehmannia glutinosa* and *Cornus officinalis*, a herbal pairing from the famous Chinese medicine prescription “Liuwei Dihuang Pill”, on adenine-induced CKD rats. The results revealed that the combination of these two herbs could modulate the composition of gut microbiota and enhance barrier function to intervene CKD. Another herb-pair containing *T. wilfordii* and *Trichosanthes kirilowii* was found to have effect on diabetic kidney disease by improving insulin resistance, inflammation, and oxidative stress (Lu et al.). A study by Liu et al. investigated the renoprotective effect of Jian-Pi-Yi-Shen (JPYS), a Chinese herbal decoction containing eight medicinal herbs, in adenine-induced CKD rat model. The results showed that JPYS alleviated renal dysfunction and fibrosis in CKD rats, and the mechanism of which might be related to the regulation of tryptophan metabolism and inhibition of aryl hydrocarbon receptor signaling activation. Finally, Zhao et al. have shown that Yishen Huashi (YSHS) granule, a herbal prescription mainly composing of roots and rhizomes of *Panax ginseng*, roots of *A. membranaceus*, rhizomes of *Atractylodes macrocephala* and *P. cocos*, ameliorated renal fibrosis and preserved the integrity of the kidney filtration barrier, and the PI3K/AKT/mTOR pathway participated in YSHS-modulated diabetic nephropathy rats. Besides, Xu et al. have conducted a bibliometric and knowledge-map analysis to analyze the research status and application of herbal medicine for the treatment of CKD. The results indicate that herbal medicine has a wide range of pharmacological activities and therapeutic value for CKD.

One article by Ni et al. has explored the nephrotoxicity of bavachin, one of the main toxic components in fruits of *Psoralea corylifolia*, and its prevention. The results confirmed that bavachin at 0.5 μM could cause obvious renal fibrosis *in vivo* and *in vitro*, and which was associated with the activation of the TGFβ1/Smad3 and Notch1/NICD signaling pathway. Furthermore, the authors discovered that ginsenoside Rb1 exerted an outstanding effect against bavachin-induced renal fibrosis via suppressing ROS-triggered endoplasmic reticulum stress. Another study by Wu et al. evaluated the efficacy and hepatotoxicity of tripterygium glycosides tablets extract from a well-known herb *T. wilfordii in vivo*. The findings showed that tripterygium glycosides exhibited clear therapeutic efficacy in nephrotic syndrome rats, but aggravated hepatotoxicity. The potential mechanism was related to the significant increase of *in vivo* exposure of the six key components in nephrotic syndrome rats. Triptolide, wilforlide A, wilforgine, wilfortrine, wilfordine and wilforine were identified as active ingredients within tripterygium glycosides, whereas triptolide, wilforgine, wilfortrine, and wilfordine were recognized as hepatotoxic components. Taken together, these studies will be useful for the clinical application of such herb.

Apart from herbal medicine for kidney disease, four articles focusing on different aspects have been included in this Research Topic. Du et al. have conducted a meta-analysis to evaluate the effectiveness of calcium dobesilate, a microcirculation-improving medication in diabetic kidney disease patients. The results showed that calcium dobesilate was effective and safe in patients with diabetic kidney disease, and the mechanism of which was involved in suppressing MAPK and chemokine signaling pathways. Feng et al. have shown that the activation of transient receptor potential ion channel 6 (TRPC6) by angiotensin II, a strong vasoconstrictor active factor induced podocyte injury and participated in proteinuria of nephrotic syndrome rat model induced by adriamycin, whereas losartan, an angiotensin receptor blocker and TRPC6-specific inhibitor SAR7334 could effectively attenuate podocyte injury and proteinuria. Liu et al. have explored the therapeutic effect of Jiawei Runjing decoction in the treatment of cryptozoospermia through two-center prospective cohort study. The findings indicated that Jiawei Runjing decoction could promote spermatogenesis in cryptozoospermia patients with varicocele, which might be closely related to improving the testicular microenvironment. Besides, Yang et al. have characterized the differences between the cold and hot properties of Chinese herbs. The results showed that the K value, a sensitive parameter could reflect the cold and hot properties of Chinese herbs.

## Conclusion

This Research Topic collects 29 articles covering a wide range of studies in the field of herbal medicine in controlling kidney disease. These findings greatly improve our understanding on the therapeutic effects and mechanisms of herbal medicine in preventing or reducing kidney damage and its related diseases. These studies also highlight unique advantages in discovering potential candidate drugs from active ingredients within herbal medicine. Thus, this topic can provide scientific evidence to support the development of herbal medicine as therapeutic strategies for the treatment of CKD.
